# Emerging roles of the P2X7 receptor in cancer pain

**DOI:** 10.1007/s11302-022-09902-1

**Published:** 2022-11-19

**Authors:** Ping Wu, Yin Wang, Yansong Liu, Yan Liu, Guohua Zhou, Xiaoqi Wu, Qingping Wen

**Affiliations:** 1 Department of Anesthesiology, The First Affiliated Hospital of Dalian Medical University, Dalian, 116011 China; 2 Anesthesiology Department, Dalian Medical University, Dalian, 116044 China; 3 Ningbo First Hospital, Ningbo, China

**Keywords:** P2X7 receptor, Cancer pain, Depression, P2X7 receptor antagonist

## Abstract

Cancer pain is the most prevalent symptom experienced by cancer patients. It substantially impacts a patient’s long-term physical and emotional health, making it a pressing issue that must be addressed. Purinergic receptor P2X7 (P2X7R) is a widely distributed and potent non-selective ATP-gated ion channel that regulates tumor proliferation, chronic pain, and the formation of inflammatory lesions in the central nervous system. P2X7R plays an essential role in cancer pain and complications related to cancer pain including depression and opioid tolerance. This review focuses on the structure and distribution of P2X7R, its role in diverse tissues in cancer pain, and the application of P2X7R antagonists in the treatment of cancer pain to propose new ideas for cancer pain management.

## Introduction

Cancer incidence and mortality rates are on the rise with population size growth, increasing aging population, and the prevalence of cancer-causing factors [1]. Cancer pain is unavoidable for patients suffering from intermediate to advanced cancer [2]. Currently, most analgesics for cancer pain follow the international three-step analgesic protocol, which begins with non-steroidal anti-inflammatory drugs (NSAIDs) and transitions to NSAIDs plus mild opioids. Finally, when the pain becomes unbearable, potent opioids are used, supplemented by other treatments including radiation therapy, nerve blocks, antiepileptics, antidepressants, and steroids [3]. A systematic review of the literature on pain in cancer patients conducted over the last 40 years revealed that, on average, 53% of cancer patients report pain, with this percentage rising to 59% in patients receiving anti-cancer treatment and 33% still experiencing pain even after cured treatment [4].

**Fig. 1:**
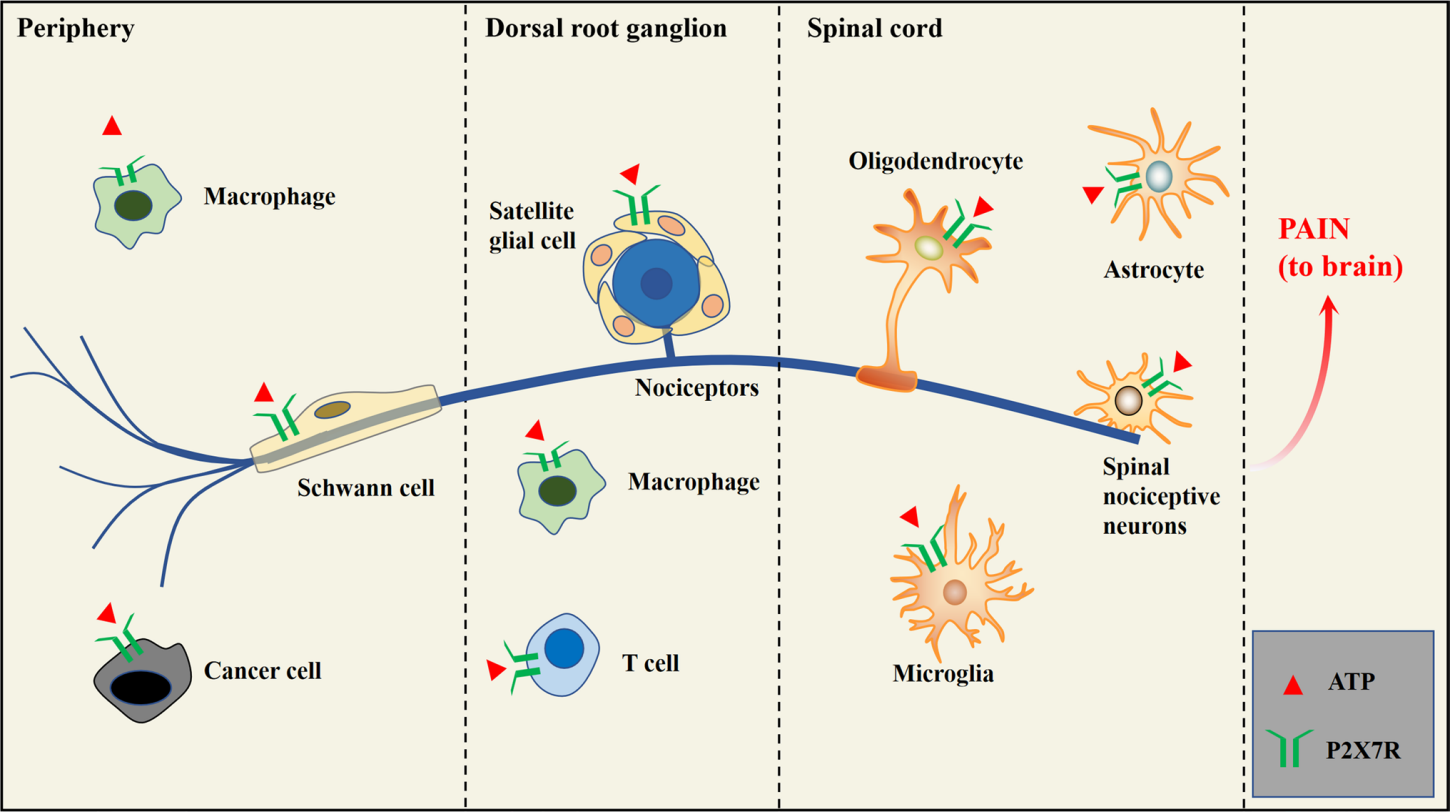
The role of P2X7R in pain generation, transmission, and sensitization

Cancer pain is chronic pain with complex and distinct mechanisms, overlapping but not identical characteristics of inflammatory and neuropathic pain [5]. Sensitizing mediators produced and secreted by tumors and associated immune cells activate peripheral injurious receptors, which travel up to the dorsal root ganglion (DRG) or trigeminal ganglion (TG), which in turn establish synapses with second-order interneurons in the dorsal horn of the spinal cord, activating spinal microglia to release inflammatory mediators [6]. These microglia interact with T cells that infiltrate the dorsal horn of the spinal cord after nerve injury and are involved in central sensitization and chronic pain [7]. Thus, due to the increased concentration of neuromodulators such as interleukin (IL)-1β, brain-derived cell growth factor (BDNF), and prostaglandin E2 in the cerebrospinal fluid, they can fine-tune excitatory or inhibitory synaptic transmission, ultimately enhancing the transmission of pain signals to the brain [8]. A large body of evidence suggests that P2X7R is a trigger for inflammatory factors and is widely distributed in tumor cells and various immune cells [9]. P2X7R can be activated by high ATP concentrations and mediates inflammatory responses by releasing pro-inflammatory cytokines such as tumor necrosis factor-α and IL-1β and activating various ion channels, G protein-coupled receptors, and tyrosine kinase receptors, thereby enhancing pain transduction and transmission [10, 11]. Significant progress has recently been made in investigating P2X7R to treat cancer pain. Moreover, many studies have revealed that negative emotions aggravate cancer pain progression, reduce patient compliance, and negatively affect cancer pain treatment [12]. The study of P2X7R in cancer pain has made significant progress in recent years, and it is likely to be an essential direction for cancer pain treatment in the future (Fig. 1).

Necrotic cells and activated immune cells release a large amount of ATP during cancer pain progression, which stimulates P2X7R-expressing macrophages, Schwann cells, and cancer cells to release pro-inflammatory factors and activate nociceptors directly. The pain signal is then transmitted to the dorsal root ganglion and cooperates with T cells to regulate neuronal plasticity and participate in central sensitization. Cells such as microglia and astrocytes that express P2X7Rs are activated at the spinal cord level, mediate inflammatory responses, and secrete neuromodulators. Finally, the pain signal is further enhanced and sent to the brain, where it produces pain sensations.

### P2X7R structure and distribution

The P2 receptor family is divided into ligand-gated ion channel P2X receptors and G protein-coupled metabolic P2Y receptors [13]. P2X is an ATP-dependent non-selective cation channel purinoceptor, and up till now, seven P2X receptors (P2X1-7) have been cloned [14]. P2X7R has a lower affinity for ATP than other P2X receptor subtypes and is not activated at normal physiological concentrations; however, when dead cells and activated immune cells are in a state of inflammation or ischemic tissue injury, they release high concentrations of ATP (> 100 µmol/L) [15]; P2X7R is activated and other forms of cation-selective channels that mediate Na^+^ and Ca^2+^ influx and K^+^ efflux, resulting in changes in cellular ion homeostasis [16, 17]. Structurally, in contrast to other P2X receptors, P2X7R has a very long intracellular C-terminus that accounts for 40% of the entire protein, which is thought to be the primary reason for its physiological functions [18]. P2X7R is widely distributed in many cell types, including (i) almost all immune cells, including dendritic cells, macrophages, monocytes, granulocytes, lymphocytes, osteoblasts, osteoclasts, and osteocytes; (ii) neural cells, such as central and spinal cord neurons, and retinal ganglion cells; (iii) glial cells, including microglia, astrocytes, Müller cells, Schwan cells, and satellite cells; and (iv) epithelial and endothelial cells [19–24]. Due to its widespread distribution, P2X7R has also been implicated in the development of several diseases., including diabetes [25], Crohn’s disease [26], depression [27], chronic pain [28], chronic neurodegenerative diseases [29], and various cancers, including breast cancer, stomach cancer, and bone metastasis [30]. P2X7R recently attracted the attention of many researchers who focused on P2X7R concerning cancer pain and found that the neurological P2X7R plays a vital role in the development of cancer pain [9]. Under physiological conditions, extracellular ATP levels are generally in the low millimolar range, so P2X7R is not activated and does not trigger a disease response. However, when the body is exposed to noxious stimuli, large amounts of ATP are released to activate P2X7R, causing changes in protein conformation, which leads to alterations in downstream cellular cascade signaling pathways, thereby promoting neurotransmitter release, neuroimmune dysregulation, and neuroinflammatory responses [31].

### Correlation of P2X7R polymorphism with cancer pain

The human *P2RX7* gene is located on chromosome 12q24.31. *P2RX7* gene has many single nucleotide polymorphism (SNP) loci that can alter the functional status of P2X7R by affecting its membrane pore-forming ability or IL-1β secretion [32]. Studies have revealed that *P2RX7* gene polymorphisms have been associated with various chronic pains, including arthralgia, diabetic neuropathic pain, and cancer pain [33]. Tao et al. compared the frequency of different alleles and the genotype distribution of selective SNPs in patients with gout and hyperuricemia. Patients with susceptible genotypes such as rs1653624 exhibited significantly higher IL-1β concentrations and prevalence than those carrying non-susceptible genotypes. The more sensitive the genotype, the more susceptible they were to the disease [34]. A recent study from the Xing laboratory investigated the relationship between SNPs and susceptibility to postherpetic neuralgia (PHN) based on different genetic models and found that rs7958311 (*P2RX7*) was significantly associated with PHN [35]. Kambur et al. used a cold pressor assay to assess the intensity and tolerance of postoperative pain in breast cancer patients. They showed that pain intensity was significantly lower in carriers of the pure-hybrid minor allele (AA) than in other genotypes. The expression of *P2RX7* SNP rs7958311 is likely to regulate pain sensitivity in humans [33].

P2X7R in rodents has an 80% homologous sequence to human P2X7R, so studying P2X7R in rats or mice can help researchers better understand the function of human P2X7R [14]. Hansen et al. observed that P2X7R-deficient C57BL/6-BALB/cj hybrid mice exhibited susceptibility to bone cancer pain (BCP). Still, later, this group in transgenic murine spinal cord monitored a shear mutant of P2X7R, P2X7 (k), which may compensate for the function of P2X7R [36]. One study used Lewis lung cancer (LLC) cells to induce BCP in *P2RX7* − / − mice on a C57BL/6 J background, showing that *P2RX7* − / − mice inoculated with LLC cells showed nociceptive sensitization compared to control mice. Although no significant differences in nociceptive performance were found between *P2RX7* − / − and wild-type mice, catwalk gait analysis parameter changes occurred earlier in *P2RX7* − / − mice than in wild-type mice, suggesting that knocking out P2X7R may accelerate the progression of cancer pain [37].

The above studies suggest that the hypothesis of P2X7R involvement in cancer pain development is inconsistent. It is speculated that this discrepancy is due to P2X7R polymorphism and related functional differences in different tissues and conditions, which should be investigated further [36].

### The role of tumor P2X7R in cancer pain

The relationship between tumor and sensory nerves is one of the most important factors influencing cancer pain in patients. Effective treatment of cancer pain can be achieved by targeting specific neuroreceptors in the tumor tissue. For example, in bone tissue, the release of Nerve growth factor (NGF) from tumor and stromal cells promoted neuropathic sprouting and neuroma formation. NGF-specific antibodies significantly reduce pain associated with metastatic bone cancer [38]. Besides, many studies have demonstrated that P2X7R is overexpressed in cancer cells and associated immune cells in a variety of malignancies, as well as that the tumor microenvironment releases large amounts of ATP, which has been shown to play an essential role in regulating tumor cell growth, apoptosis, migration, and erosion by activating P2X7R in tumor tissues [39–41]. However, P2X7R acts as a trigger for inflammatory cytokine release, but no study has been reported on the association of P2X7R in the tumor microenvironment with cancer pain. Our study used Walker 256 breast cancer cells to create a rat BCP model. X-rays revealed that Brilliant Blue G (BBG) could not ameliorate the tumor damage to the bone. In vitro experiments did not detect any expression of P2X7R in Walker 256 breast cancer cells. As a result, it is speculated that P2X7R expression and the therapeutic effect of BBG may be related to the specificity of inoculated cells [42].

Currently, animal experiments used to study the mechanism of cancer pain primarily use BCP models. However, bone tissue detection is relatively difficult due to the complexity of the bone microenvironment. We believe that with the progress of bone tissue detection technology, the research prospect for bone tumor microenvironment P2X7R and pain will become very broad. Furthermore, given that specific P2X7R antagonists effectively inhibit the growth and migration of tumors such as breast [43] and colon [44] cancers in various animal models and clinical trials, we hypothesized that P2X7R is likely to be a promising pharmacological target for treating cancer and cancer pain.

### Involvement of spinal microglia expressed P2X7R in cancer pain

Microglia are a macrophage-like cell population that resides in the central nervous system (CNS) and play a crucial role in maintaining tissue homeostasis [45]. Numerous studies have revealed that P2X7R is predominantly expressed in spinal cord segments on microglia in the dorsal horn of the spinal cord and that P2X7R activation promotes the release of large amounts of inflammatory mediators from microglia in response to adverse external stimuli, resulting in the maintenance of pain and central sensitization [46].

According to literature, P2X7R modulation of microglia polarization toward M1 pro-inflammatory type has been observed in chronic pain models such as neuropathic pain and ischemia–reperfusion injury pain [47]. Administration of P2X7R antagonists can inhibit microglia polarization to the pro-inflammatory M1 type and promote their polarization to the anti-inflammatory M2 type, effectively relieving pain [48]. Additionally, our experimental group used Walker 256 rat breast cancer cell line and discovered that P2X7R was involved in the polarization of microglia in the dorsal horn of the spinal cord in a rat breast cancer BCP model for the first time. However, it is debatable whether P2X7R is involved in the entire process of BCP development and progression in the dorsal horn microglia of the spinal cord. Although Yang et al. demonstrated that P2X7R is involved in microglia activation in a rat model of breast cancer–induced BCP, it is active during the maintenance phase of BCP, not during the initial phase [10]. Falk et al. demonstrated that intrathecal administration of P2X7R-specific inhibitor A839977 significantly decreased pain in the initial and maintenance phases of a BCP model in rats injected with MRMT-1 breast cancer cells [49]. Our group used Walker 256 rat breast cancer cell line. It indicated that although microglia were not activated until the 14th day after tumor inoculation, the administration of P2X7R-specific inhibitor BBG was effective in relieving BCP at the onset of pain, implying that P2X7R is involved in the development and progression of BCP [42]. Interestingly, microglia were not significantly activated in the early stages of BCP. Microglia activation and nociceptive sensitization are not entirely synchronous, and the precise mechanism needs to be investigated in depth. Falk et al. demonstrated that administering therapeutic doses of P2X7R antagonist A-839977 was ineffective at suppressing BCP and that high doses of A-839977 exacerbated the pain [49]. Despite this, many reports indicate that microglia are not involved in BCP formation, such as the study by Ducourneau et al., who created a rat model of breast cancer BCP by injecting MRMT-1 cells and examined the expression of microglia markers IBA-1 and OX-42 and found no evidence of microglia activation [50].

The above results suggest that the role of P2X7R expressed by microglia in the dorsal horn of the spinal cord in a BCP model is influenced by the tumor cells used to construct the BCP model species of experimental animals and the research method. Therefore, the role of P2X7R in the inoculation of different tumor cells and different species of BCP models needs to be further investigated.

### Role of P2X7R in depression due to cancer pain

Cancer pain and depression have become two of the most prevalent and burdensome challenges cancer patients face. Numerous studies show that cancer pain and depression may share the same signaling pathways and neurotransmitters [51]. Long-term untreated cancer pain can result in severe mental disorders, and this secondary mood disorder complicates and aggravates pain treatment [52, 53]. As a result, identifying typical receptors that connect the two is likely to be an effective target for the future treatment of cancer pain with depression-related disorders.

Recent epidemiological studies have established that the human *P2RX7* gene, located on chromosome 12q24.31, is a susceptibility locus for affective disorders [54]. P2X7R is strongly correlated with neuropsychiatric disorders such as depression and plays an important role in central neuroinflammatory pathways [55]. Liu et al. confirmed that pain-induced depressed rats had significantly higher hippocampal P2X7R expression than controls [56]. Wang et al. further demonstrated that hippocampal P2X7R is vital in developing nociceptive transmission and depressive behavior in rats with diabetic neuropathic pain combined with depression and reducing IL-1β production in hippocampal regions. Administering inhibitors of P2X7R can effectively alleviate pain and depressive behavior [57]. Recent research has demonstrated that P2X7R/PRG-1/PP2A plays an important role in regulating synaptic plasticity in the hippocampus of rats with BCP in the development of cancer pain and depressive behavior and that both P2X7R inhibitors and PRG-1 agonists by injection into the hippocampal region can effectively alleviate cancer pain and depressive-like behavior in rats [56]. P2X7R was found to be upregulated in microglia in the rostral ventromedial medulla (RVM) in a rat model of BCP, and microscopic injection of BBG or P2X7R siRNA into RVM reduced the expression of D-type serine and 5-hydroxytryptamine, effectively relieving pain and depression-like behavior [58]. In recent literature, it was reported that in a rat model of tongue cancer pain, P2X7R was predominantly expressed in microglia in the caudal subnucleus of the trigeminal spinal tract nucleus, and the administration of P2X7R-specific antagonist BBG inhibited pannexin 1 /IL-1β and thus effectively relieved tongue cancer pain [59].

Thus, P2X7R is involved in the onset and development of cancer pain and psychiatric disorders such as depression via neuroimmune and neuroinflammatory mechanisms, and P2X7R is likely to exacerbate cancer pain and depressive comorbidities by activating the central downstream emmetropic system. Therefore, P2X7R appears to be a potentially useful therapeutic target for cancer pain and secondary depression.

### Role of P2X7R in morphine analgesic tolerance and nociceptive hyperalgesia

Opioids (e.g., morphine, fentanyl, and oxycodone) commonly treat moderate to severe cancer pain [60]. However, as the duration of use prolongs, adverse effects such as dependence, tolerance, and nociceptive hypersensitivity may gradually manifest [61]. There is mounting evidence that P2X7R is involved in developing morphine analgesic tolerance and nociceptive hyperalgesia, which is mainly associated with the fact that long-term high-dose morphine application causes upregulation of P2X7R expression in the spinal cord and hippocampus, and inhibiting P2X7R expression inhibits the development of morphine analgesic tolerance [62]. Zhou et al. demonstrated that P2X7R inhibitors or siRNA effectively reversed morphine tolerance by inhibiting the activation of spinal microglia [63]. The midbrain’s periaqueductal gray matter (PAG) is a critical component of the brain’s endogenous analgesic system. The analgesic effect of morphine can be attenuated by injecting oligonucleotides of P2X7R into PAG, whereas injecting 2′ (3′)-O-(4-benzoylbenzoyl)ATP (BzATP) into PAG exacerbates morphine tolerance development [64]. Chen et al. proposed for the first time that ATP is involved in the onset and development of morphine analgesic tolerance by activating P2X7R-containing microglia to release IL-18 thereby initiating a downstream cascade response. D-serine, an endogenous ligand for the NMDAR glycine site, is generally released by microglia and astrocytes and is closely associated with synapse formation and neuronal development. This experiment also demonstrated that D-amino acid oxygenase (DAAO) attenuated morphine tolerance [65]. Similar to the spinal cord, D-serine content and the serine racemase protein expression level were significantly upregulated in the ventrolateral region of the midbrain periaqueductal gray matter in morphine-tolerant rats. The activation of P2X7R is an important prerequisite for D-serine release [65], and the injection of D-amino acid oxidase or antisense oligonucleotides targeting P2X7R in the ventral lateral region of the gray matter surrounding the midbrain conduction can significantly alleviate the progression of morphine tolerance [66].

Thus, P2X7R is critical in promoting morphine tolerance in animal cancer pain models. The specific mechanism by which P2X7R contributes to this process has research significance and clinical application value, as cancer pain requires long-term high-dose morphine application for all pain types and warrants in-depth study [67]. The recent study of P2X7R and opioid tolerance is limited to morphine. Still, as opioids continue to evolve, other opioids are commonly used in treating moderate to severe cancer pain. Whether the formation and development of tolerance to these opioids are related to P2X7R remains unknown.

### P2X7R antagonists in the treatment of cancer pain

P2X7R has been the most popular subtype of the P2X family, and P2X7R antagonists are increasingly being used to treat various pain types (Table 1) [68]. Numerous studies have indicated that P2X7 knockout mice effectively resist inflammatory and neuropathic pain, implying that P2X7R may be an important target for pain treatment [36]. P2X7R ligand research advances have recently identified several different types of P2X7R antagonists, which are divided into the following six major groups; BBG, a polysulfone acid dye, is a potent noncompetitive inhibitor of rat and human P2X7R. It can alleviate bone cancer pain mainly by inhibiting glial cell activation and inflammatory response. In addition, BBG can also relieve pain-related depressive behaviors [69]. The second group is some natural products and derived complexes [70]. It has been demonstrated that 1 and 10 µM emodin reduced ATP-induced increase of intracellular Ca^2+^ concentration by 35% and 60%, respectively, thereby inhibiting P2X7R function [71, 72]. Specifically, ROS, NO, and IL-1β will all be reduced by colchicine. The third group is a newly synthesized molecule with various structural and chemical conformation types, including A438079, A740003, A804598, AZ10606120, AZ11645373, and KN-62. Some of these antagonists may relieve not only cancer pain, but other types of neuropathic pain as well as pain-related mood disorders. As for their mechanisms in treating cancer pain, one can find that these antagonists mainly work by regulating the inflammatory response and ion transport. Among them, A438079, A804598, and AZ1164537 can inhibit microglia function and inflammatory response in the process of reliving cancer pain [73–75] while A740003 and AZ10606120 can alleviate cancer pain by reducing calcium influx [76, 77]. Additionally, KN-62 can not only inhibit ATP-dependent Ca^2+^ influx and ethidium bromide uptake, but also reduce the secretion of IL-1β.

**Table 1:** Research status of P2X7R in the mechanism of cancer pain

Antagonists	Chemical formula	Weight	Function	Mechanism	References
BBG	C_47_H_48_N_3_NaO_7_S_2_	854.02	BBG reduces glial cell activation and pain caused by bone cancer	By inhibiting the release of IL-1β and IL-18, BBG reduces inflammation, pain, and depressive behaviors	[27, 58, 78]
Emodin	C_15_H_10_O_5_	270.24	Emodin reduces P2X7R-dependent signaling and invasiveness of cancer cells in vitro and in vivo	Emodin inhibits P2X7R activation, Ca^2+^ increase, IL-1β release, ROS production, and phagocytosis	[71, 79]
Colchicine	C_22_H_25_NO_6_	399.44	Colchicine has a good therapeutic effect on inflammatory diseases such as gouty arthritis, osteoarthritis, and pericarditis	Colchicine reduces reactive oxygen species (ROS) formation and release of nitric oxide (NO), interleukin (IL)-1β	[72, 80]
A438079	C_13_H_9_C_l2_N_5_	306.15	A438079 plays a key role in bone cancer–induced pain (including thermal hyperalgesia and mechanical allodynia) and depression-like behavior in rats	A438079 reduces the expression and apoptosis of IL-1β, IL-18, P2X7R, ox-LDL, CXCL16, Bax, caspase-3, and NLRP3	[56, 74]
A740003	C_26_H_30_N_6_O_3_	474.55	In rats with BCP, pretreatment with A-740003 injection into the periaqueductal gray of the midbrain can significantly antagonize tramadol’s analgesic effect on central opioid receptor analgesia	A740003 significantly inhibits the increase of intracellular calcium ion concentration and effectively reduces the number of apoptotic chondrocytes	[77, 81]
A804598	C_19_H_17_N_5_	315.37	A804598 may treat neuroinflammatory disease progression and relieve pain by protecting microglia	A804598 inhibits the death of microglia and can reduce the expression of pro-inflammatory	[73, 82]
AZ10606120	C_25_H_36_C_l2_N_4_O_2_	495.49	AZ10606120 may regulate neuropathic pain and inhibit tumor cell proliferation and migration	AZ10606120 inhibits P2X7R-mediated accumulation of Yo-Pro-1 in cells and calcium influx	[83, 84]
AZ11645373	C_24_H_21_N_3_O_5_S	463.51	AZ11645373 inhibits human P2X7R with IC50 of ~ 90 nM and has therapeutic effects on chronic pain, mood disorders, and cancer	Treatment of macrophages with AZ11645373 reduces NLRP3 inflammasome-dependent IL-1β secretion	[75, 85]
KN-62	C_38_H_35_N_5_O_6_S_2_	721.84	KN-62 can reduce the expression of tolerance to nicotine-induced analgesia in mice in a dose-dependent manner	KN-62 inhibits ATP-dependent Ca^2+^ influx across the plasma membrane, ethidium bromide uptake, and secretion of the cytokine IL-1β	[86, 87]

## Progress and prospect

P2X7R is another potential therapeutic target worth exploring for cancer and pain. P2X7R is important in chronic pain because it promotes cancer pain and plays a regulatory role in tumor development. Despite the controversy surrounding P2X7R, many studies support the important role of P2X7R in cancer pain and demonstrate significant potential in pharmacological studies. Based on our review and summary of the literature, we assume that P2X7R is involved in the development and progression of cancer pain at the peripheral and central nervous system levels and in the development and maintenance of psychiatric disorders such as depression via neuroimmune and neuroinflammatory mechanisms. Cancer pain is most prone to opioid tolerance, so the role of P2X7R in nociceptive sensitization for cancer pain treatment is critical. An in-depth investigation of the mechanism of action of P2X7R can help reveal the pathogenesis of cancer pain and affective depressive disorder in tumor patients, with far-reaching implications for identifying novel therapeutic targets.

## Data Availability

All of the material is owned by the authors and/or no permissions are required.

## References

[1] DeSantis CE, Miller KD, Dale W, Mohile SG, Cohen HJ, Leach CR, Goding Sauer A, Jemal A (2019). Siegel RL (2019) Cancer statistics for adults aged 85 years and older. CA Cancer J Clin.

[2] van den Beuken-van Everdingen MH, Hochstenbach LM, Joosten EA, Tjan-Heijnen VC, Janssen DJ (2016). Update on prevalence of pain in patients with cancer: systematic review and meta-analysis. J Pain Symptom Manage.

[3] Zech DFJ, Grond S, Lynch J, Hertel D, Lehmann KA (1995). Validation of World Health Organization Guidelines for cancer pain relief: a 10-year prospective study. Pain.

[4] van den Beuken-van Everdingen MH, de Rijke JM, Kessels AG, Schouten HC, van Kleef M, Patijn J (2007). Prevalence of pain in patients with cancer: a systematic review of the past 40 years. Ann Oncol.

[5] Kane CM, Hoskin P, Bennett MI (2015). Cancer induced bone pain. BMJ.

[6] Zhu G, Dong Y, He X, Zhao P, Yang A, Zhou R, Ma J, Xie Z, Song XJ (2016). Radiotherapy suppresses bone cancer pain through inhibiting activation of cAMP signaling in rat dorsal root ganglion and spinal cord. Mediators Inflamm.

[7] Costigan M, Moss A, Latremoliere A, Johnston C, Verma-Gandhu M, Herbert TA, Barrett L, Brenner GJ, Vardeh D, Woolf CJ (2009). T-cell infiltration and signaling in the adult dorsal spinal cord is a major contributor to neuropathic pain-like hypersensitivity. J Neurosci.

[8] Ji RR, Chamessian A, Zhang YQ (2016). Pain regulation by non-neuronal cells and inflammation. Science.

[9] Falk S, Appel CK, Bennedbaek HB, Al-Dihaissy T, Unger A, Dinkel K, Heegaard AM (2019). Chronic high dose P2X7 receptor inhibition exacerbates cancer-induced bone pain. Eur J Pharmacol.

[10] Yang Y, Li H, Li TT, Luo H, Gu XY, Lu N, Ji RR, Zhang YQ (2015). Delayed activation of spinal microglia contributes to the maintenance of bone cancer pain in female Wistar rats via P2X7 receptor and IL-18. J Neurosci.

[11] Xu Y, Liu J, He M, Liu R, Belegu V, Dai P, Liu W, Wang W, Xia QJ, Shang FF (2016). Mechanisms of PDGF siRNA-mediated inhibition of bone cancer pain in the spinal cord. Sci Rep.

[12] Kwekkeboom K, Zhang Y, Campbell T, Coe CL, Costanzo E, Serlin RC, Ward S (2018). Randomized controlled trial of a brief cognitive-behavioral strategies intervention for the pain, fatigue, and sleep disturbance symptom cluster in advanced cancer. Psychooncology.

[13] Pannicke T, Fischer W, Biedermann B, Schadlich H, Grosche J, Faude F, Wiedemann P, Allgaier C, Illes P, Burnstock G (2000). P2X7 receptors in Muller glial cells from the human retina. J Neurosci.

[14] Illes P, Muller CE, Jacobson KA, Grutter T, Nicke A, Fountain SJ, Kennedy C, Schmalzing G, Jarvis MF, Stojilkovic SS (2021). Update of P2X receptor properties and their pharmacology: IUPHAR Review 30. Br J Pharmacol.

[15] Pellegatti P, Raffaghello L, Bianchi G, Piccardi F, Pistoia V, Di Virgilio F (2008). Increased level of extracellular ATP at tumor sites: in vivo imaging with plasma membrane luciferase. PLoS One.

[16] McLarnon JG (2005). Purinergic mediated changes in Ca2+ mobilization and functional responses in microglia: effects of low levels of ATP. J Neurosci Res.

[17] Liang X, Samways DS, Wolf K, Bowles EA, Richards JP, Bruno J, Dutertre S, DiPaolo RJ, Egan TM (2015). Quantifying Ca2+ current and permeability in ATP-gated P2X7 receptors. J Biol Chem.

[18] Kopp R, Krautloher A, Ramirez-Fernandez A, Nicke A (2019). P2X7 interactions and signaling - making head or tail of it. Front Mol Neurosci.

[19] He X, Wan J, Yang X, Zhang X, Huang D, Li X, Zou Y, Chen C, Yu Z, Xie L et al (2021) Bone marrow niche ATP levels determine leukemia-initiating cell activity via P2X7 in leukemic models. J Clin Invest 131(4):10.1172/JCI140242 10.1172/JCI140242 PMC7880412 33301426

[20] Illes P, Rubini P, Ulrich H, Zhao Y, Tang Y (2020) Regulation of microglial functions by purinergic mechanisms in the healthy and diseased CNS. Cells 9(5):10.3390/cells9051108 10.3390/cells9051108 PMC7290360 32365642

[21] Chen Q, Wu H, Tao J, Liu C, Deng Z, Liu Y, Chen G, Liu B, Xu C (2017). Effect of naringin on gp120-induced injury mediated by P2X7 receptors in rat primary cultured microglia. PLoS One.

[22] Ebeling G, Blasche R, Hofmann F, Augstein A, Kasper M, Barth K (2014). Effect of P2X7 receptor knockout on AQP-5 expression of type I alveolar epithelial cells. PLoS One.

[23] Vessey KA, Fletcher EL (2012). Rod and cone pathway signalling is altered in the P2X7 receptor knock out mouse. PLoS One.

[24] Sanderson J, Dartt DA, Trinkaus-Randall V, Pintor J, Civan MM, Delamere NA, Fletcher EL, Salt TE, Grosche A, Mitchell CH (2014). Purines in the eye: recent evidence for the physiological and pathological role of purines in the RPE, retinal neurons, astrocytes, Muller cells, lens, trabecular meshwork, cornea and lacrimal gland. Exp Eye Res.

[25] Lu Z, Yao Y, Wang J, Peng JY (2021). Dioscin ameliorates diabetes cognitive dysfunction via adjusting P2X7R/NLRP3 signal. Int Immunopharmacol.

[26] Zhang J, Wang XJ, Wu LJ, Yang L, Yang YT, Zhang D, Hong J, Li XY, Dong XQ, Guo XC (2021). Herb-partitioned moxibustion alleviates colonic inflammation in Crohn’s disease rats by inhibiting hyperactivation of the NLRP3 inflammasome via regulation of the P2X7R-Pannexin-1 signaling pathway. PLoS One.

[27] Yue N, Huang H, Zhu X, Han Q, Wang Y, Li B, Liu Q, Wu G, Zhang Y, Yu J (2017). Activation of P2X7 receptor and NLRP3 inflammasome assembly in hippocampal glial cells mediates chronic stress-induced depressive-like behaviors. J Neuroinflammation.

[28] Wu Q, Yue J, Lin L, Yu X, Zhou Y, Ying X, Chen X, Tu W, Lou X, Yang G (2021). Electroacupuncture may alleviate neuropathic pain via suppressing P2X7R expression. Mol Pain.

[29] Illes P (2020) P2X7 receptors amplify CNS damage in neurodegenerative diseases. Int J Mol Sci 21(17):10.3390/ijms21175996 10.3390/ijms21175996 PMC7504621 32825423

[30] Drill M, Jones NC, Hunn M, O'Brien TJ, Monif M (2021). Antagonism of the ATP-gated P2X7 receptor: a potential therapeutic strategy for cancer. Purinergic Signal.

[31] Rodrigues RJ, Tome AR, Cunha RA (2015). ATP as a multi-target danger signal in the brain. Front Neurosci.

[32] Stokes L, Fuller SJ, Sluyter R, Skarratt KK, Gu BJ, Wiley JS (2010). Two haplotypes of the P2X(7) receptor containing the Ala-348 to Thr polymorphism exhibit a gain-of-function effect and enhanced interleukin-1beta secretion. FASEB J.

[33] Kambur O, Kaunisto MA, Winsvold BS, Wilsgaard T, Stubhaug A, Zwart JA, Kalso E, Nielsen CS (2018). Genetic variation in P2RX7 and pain tolerance. Pain.

[34] Tao JH, Cheng M, Tang JP, Dai XJ, Zhang Y, Li XP, Liu Q, Wang YL (2017). Single nucleotide polymorphisms associated with P2X7R function regulate the onset of gouty arthritis. PLoS One.

[35] Xing X, Bai Y, Sun K, Chen Q, Huang H, Qiu W, Yan M (2020). Identification of candidate genes associated with postherpetic neuralgia susceptibility. Pain Physician.

[36] Hansen RR, Nielsen CK, Nasser A, Thomsen SIM, Eghorn LF, Pham Y, Schulenburg C, Syberg S, Ding M, Stojilkovic SS (2011). P2X7 receptor-deficient mice are susceptible to bone cancer pain. Pain.

[37] Zhao X, Liu HZ, Zhang YQ (2016). Effect of P2X7 receptor knock-out on bone cancer pain in mice. Sheng Li Xue Bao.

[38] Halvorson KG, Kubota K, Sevcik MA, Lindsay TH, Sotillo JE, Ghilardi JR, Rosol TJ, Boustany L, Shelton DL, Mantyh PW (2005). A blocking antibody to nerve growth factor attenuates skeletal pain induced by prostate tumor cells growing in bone. Cancer Res.

[39] Qin J, Zhang X, Tan B, Zhang S, Yin C, Xue Q, Zhang Z, Ren H, Chen J, Liu M (2020). Blocking P2X7-mediated macrophage polarization overcomes treatment resistance in lung cancer. Cancer Immunol Res.

[40] Tian J, Yu T, Xu Y, Pu S, Lv Y, Zhang X, Du D (2018). Swimming training reduces neuroma pain by regulating neurotrophins. Med Sci Sports Exerc.

[41] Di Virgilio F, Sarti AC, Falzoni S, De Marchi E, Adinolfi E (2018). Extracellular ATP and P2 purinergic signalling in the tumour microenvironment. Nat Rev Cancer.

[42] Wu P, Zhou G, Wu X, Lv R, Yao J, Wen Q (2022). P2X7 receptor induces microglia polarization to the M1 phenotype in cancer-induced bone pain rat models. Mol Pain.

[43] Xia J, Yu X, Tang L, Li G, He T (2015). P2X7 receptor stimulates breast cancer cell invasion and migration via the AKT pathway. Oncol Rep.

[44] Di Cesare ML, Marcoli M, Micheli L, Zanardelli M, Maura G, Ghelardini C, Cervetto C (2015). Oxaliplatin evokes P2X7-dependent glutamate release in the cerebral cortex: A pain mechanism mediated by Pannexin 1. Neuropharmacology.

[45] Borst K, Dumas AA, Prinz M (2021). Microglia: immune and non-immune functions. Immunity.

[46] Sperlagh B, Illes P (2014). P2X7 receptor: an emerging target in central nervous system diseases. Trends Pharmacol Sci.

[47] Gui X, Wang H, Wu L, Tian S, Wang X, Zheng H, Wu W (2020). Botulinum toxin type A promotes microglial M2 polarization and suppresses chronic constriction injury-induced neuropathic pain through the P2X7 receptor. Cell Biosci.

[48] Ren WJ, Illes P (2022). Involvement of P2X7 receptors in chronic pain disorders. Purinergic Signal.

[49] Falk S, Schwab SD, Frosig-Jorgensen M, Clausen RP, Dickenson AH, Heegaard AM (2015). P2X7 receptor-mediated analgesia in cancer-induced bone pain. Neuroscience.

[50] Ducourneau VRR, Dolique T, Hachem-Delaunay S, Miraucourt LS, Amadio A, Blaszczyk L, Jacquot F, Ly J, Devoize L, Oliet SHR (2014). Cancer pain is not necessarily correlated with spinal overexpression of reactive glia markers. Pain.

[51] Sheng J, Liu S, Wang Y, Cui R, Zhang X (2017). The link between depression and chronic pain: neural mechanisms in the brain. Neural Plast.

[52] Michaelides A, Zis P (2019). Depression, anxiety and acute pain: links and management challenges. Postgrad Med.

[53] Brinkers M, Pfau G, Toepffer AM, Meyer F, Kretzschmar MA (2021). The incidence of mental disorders increases over time in patients with cancer pain: data from a retrospective cohort study. Pain Res Manag.

[54] Barden N, Harvey M, Gagne B, Shink E, Tremblay M, Raymond C, Labbe M, Villeneuve A, Rochette D, Bordeleau L (2006). Analysis of single nucleotide polymorphisms in genes in the chromosome 12Q24.31 region points to P2RX7 as a susceptibility gene to bipolar affective disorder. Am J Med Genet B Neuropsychiatr Genet.

[55] Silberstein S, Liberman AC, Dos Santos Claro PA, Ugo MB, Deussing JM, Arzt E (2021). Stress-related brain neuroinflammation impact in depression: role of the corticotropin-releasing hormone system and P2X7 receptor. NeuroImmunoModulation.

[56] Liu X, Xie Z, Li S, He J, Cao S, Xiao Z (2021). PRG-1 relieves pain and depressive-like behaviors in rats of bone cancer pain by regulation of dendritic spine in hippocampus. Int J Biol Sci.

[57] Wang D, Wang H, Gao H, Zhang H, Zhang H, Wang Q, Sun Z (2020). P2X7 receptor mediates NLRP3 inflammasome activation in depression and diabetes. Cell Biosci.

[58] Huang ZX, Lu ZJ, Ma WQ, Wu FX, Zhang YQ, Yu WF, Zhao ZQ (2014). Involvement of RVM-expressed P2X7 receptor in bone cancer pain: mechanism of descending facilitation. Pain.

[59] Koyama R, Iwata K, Hayashi Y, Hitomi S, Shibuta I, Furukawa A, Asano S, Kaneko T, Yonehara Y, Shinoda M (2021) Pannexin 1-mediated ATP signaling in the trigeminal spinal subnucleus caudalis is involved in tongue cancer pain. Int J Mol Sci 22(21):10.3390/ijms222111404 10.3390/ijms222111404 PMC8584113 34768835

[60] Wiffen PJ, Wee B, Derry S, Bell RF, Moore RA (2017). Opioids for cancer pain - an overview of Cochrane reviews. Cochrane Database Syst Rev.

[61] Mercadante S, Arcuri E, Santoni A (2019). Opioid-induced tolerance and hyperalgesia. CNS Drugs.

[62] Wang H, Zhang Y, Ma X, Wang W, Xu X, Huang M, Xu L, Shi H, Yuan T, Jiang W (2020). Spinal TLR4/P2X7 receptor-dependent NLRP3 inflammasome activation contributes to the development of tolerance to morphine-induced antinociception. J Inflamm Res.

[63] Chu YX, Zhang Y, Zhang YQ, Zhao ZQ (2010). Involvement of microglial P2X7 receptors and downstream signaling pathways in long-term potentiation of spinal nociceptive responses. Brain Behav Immun.

[64] Xiao Z, Li YY, Sun MJ (2015). Activation of P2X7 receptors in the midbrain periaqueductal gray of rats facilitates morphine tolerance. Pharmacol Biochem Behav.

[65] Chen ML, Cao H, Chu YX, Cheng LZ, Liang LL, Zhang YQ, Zhao ZQ (2012). Role of P2X7 receptor-mediated IL-18/IL-18R signaling in morphine tolerance: multiple glial-neuronal dialogues in the rat spinal cord. J Pain.

[66] Cao S, Xiao Z, Sun M, Li Y (2016) D-serine in the midbrain periaqueductal gray contributes to morphine tolerance in rats. Mol Pain 1210.1177/1744806916646786 10.1177/1744806916646786 PMC4956000 27175014

[67] Grant M, Ugalde A, Vafiadis P, Philip J (2015). Exploring the myths of morphine in cancer: views of the general practice population. Support Care Cancer.

[68] Gelin CF, Bhattacharya A, Letavic MA (2020). P2X7 receptor antagonists for the treatment of systemic inflammatory disorders. Prog Med Chem.

[69] Custer EE, Knott TK, Ortiz-Miranda S, Lemos JR (2018) Effects of calcium and sodium on ATP-induced vasopressin release from rat isolated neurohypophysial terminals. J Neuroendocrinol e12605. 10.1111/jne.12605 10.1111/jne.12605 PMC6215752 29729039

[70] Faria R, Ferreira L, Bezerra R, Frutuoso V, Alves L (2012). Action of natural products on p2 receptors: a reinvented era for drug discovery. Molecules.

[71] Jelassi B, Anchelin M, Chamouton J, Cayuela ML, Clarysse L, Li J, Gore J, Jiang LH, Roger S (2013). Anthraquinone emodin inhibits human cancer cell invasiveness by antagonizing P2X7 receptors. Carcinogenesis.

[72] Marques-da-Silva C, Chaves MM, Castro NG, Coutinho-Silva R, Guimaraes MZ (2011). Colchicine inhibits cationic dye uptake induced by ATP in P2X2 and P2X7 receptor-expressing cells: implications for its therapeutic action. Br J Pharmacol.

[73] Liu Y, Wu Y, Gu S, Yin Q, Li H, Wang J, Geng D, Xu Y (2020). The P2X7 receptor (P2X7R)-specific antagonist A804598 inhibits inflammatory reaction in human fibroblast-like synoviocytes. Am J Transl Res.

[74] Jiang W, Li M, He F, Zhou S, Zhu L (2017). Targeting the NLRP3 inflammasome to attenuate spinal cord injury in mice. J Neuroinflammation.

[75] Rosli S, Kirby FJ, Lawlor KE, Rainczuk K, Drummond GR, Mansell A, Tate MD (2019). Repurposing drugs targeting the P2X7 receptor to limit hyperinflammation and disease during influenza virus infection. Br J Pharmacol.

[76] Allsopp RC, Dayl S, Schmid R, Evans RJ (2017). Unique residues in the ATP gated human P2X7 receptor define a novel allosteric binding pocket for the selective antagonist AZ10606120. Sci Rep.

[77] Li Z, Huang Z, Zhang H, Lu J, Wei Y, Yang Y, Bai L (2021). IRE1-mTOR-PERK axis coordinates autophagy and ER stress-apoptosis induced by P2X7-mediated Ca(2+) influx in osteoarthritis. Front Cell Dev Biol.

[78] Zhu Y, Zhang S, Wu Y, Wang J (2021). P2X7 receptor antagonist BBG inhibits endoplasmic reticulum stress and pyroptosis to alleviate postherpetic neuralgia. Mol Cell Biochem.

[79] Zhu S, Wang Y, Wang X, Li J, Hu F (2014). Emodin inhibits ATP-induced IL-1beta secretion, ROS production and phagocytosis attenuation in rat peritoneal macrophages via antagonizing P2X(7) receptor. Pharm Biol.

[80] Leung YY, Yao Hui LL, Kraus VB (2015). Colchicine–update on mechanisms of action and therapeutic uses. Semin Arthritis Rheum.

[81] Li P, Zhang Q, Xiao Z, Yu S, Yan Y, Qin Y (2018). Activation of the P2X7 receptor in midbrain periaqueductal gray participates in the analgesic effect of tramadol in bone cancer pain rats. Mol Pain.

[82] He Y, Taylor N, Fourgeaud L, Bhattacharya A (2017). The role of microglial P2X7: modulation of cell death and cytokine release. J Neuroinflammation.

[83] Brisson L, Chadet S, Lopez-Charcas O, Jelassi B, Ternant D, Chamouton J, Lerondel S, Le Pape A, Couillin I, Gombault A et al (2020) P2X7 receptor promotes mouse mammary cancer cell invasiveness and tumour progression, and is a target for anticancer treatment. Cancers (Basel) 12 (9):10.3390/cancers12092342 10.3390/cancers12092342 PMC7565976 32825056

[84] Hempel C, Norenberg W, Sobottka H, Urban N, Nicke A, Fischer W, Schaefer M (2013). The phenothiazine-class antipsychotic drugs prochlorperazine and trifluoperazine are potent allosteric modulators of the human P2X7 receptor. Neuropharmacology.

[85] Caseley EA, Muench SP, Baldwin SA, Simmons K, Fishwick CW, Jiang LH (2015). Docking of competitive inhibitors to the P2X7 receptor family reveals key differences responsible for changes in response between rat and human. Bioorg Med Chem Lett.

[86] Baraldi PG, del Carmen NM, Morelli A, Falzoni S, Di Virgilio F, Romagnoli R (2003). Synthesis and biological activity of N-arylpiperazine-modified analogues of KN-62, a potent antagonist of the purinergic P2X7 receptor. J Med Chem.

[87] Damaj MI (2005). Calcium-acting drugs modulate expression and development of chronic tolerance to nicotine-induced antinociception in mice. J Pharmacol Exp Ther.

